# Characteristics of and treatment outcomes in rifampicin-intolerant patients

**DOI:** 10.5588/ijtldopen.23.0466

**Published:** 2024-04-01

**Authors:** R. Mangat, S.K. Brode, H.K. Mah, M.S. Brar, N.F. Sabur

**Affiliations:** ^1^ West Park Healthcare Centre, York, ON,; ^2^ Department of Respirology, Toronto Western Hospital, West Park Healthcare Centre, Toronto, ON,; ^3^ Departments of Medicine, and; ^4^ Surgery, University of Toronto, Toronto, ON,; ^5^ Department of Respirology, St. Michael’s Hospital, West Park Healthcare Centre, Toronto, ON, Canada

**Keywords:** tuberculosis treatment, treatment intolerance, RIF, rifabutin, adverse effects

## Abstract

**BACKGROUND:**

Rifampicin (RIF) is considered the backbone of TB treatment, but adverse effects often limit its use.

**METHODS:**

This retrospective cohort study examined patients treated for TB disease at our institution, and compared those who received RIF to those who were intolerant to RIF.

**RESULTS:**

A total of 829 patients were included. Seventy-six patients (9%) were intolerant to RIF. Patients with RIF intolerance were significantly older (median age: 67 years, IQR 50–78 vs. 48 years, IQR 31–70; *P* < 0.0001), and were more likely to be female (57% vs. 41%; *P* = 0.01) and have concurrent diabetes mellitus (37.3% vs. 19%; *P* < 0.0001) compared to those who tolerated RIF. RIF intolerance was most commonly due to transaminitis (25%), cytopenia (14.5%), rash (17.1%) and gastro-intestinal intolerance (7.8%). Twenty patients were subsequently challenged with rifabutin, and this was successful in 70%. The mean treatment duration was significantly longer in patients who were intolerant to RIF (335 vs. 270 days; *P* < 0.001). There was no significant difference in treatment outcomes.

**CONCLUSION:**

RIF intolerance is more common in older patients, females, and those with concurrent diabetes mellitus. Patients who could not tolerate RIF had a longer duration of therapy, but no difference in treatment outcomes. When attempted, rifabutin was well tolerated in most patients with a previous RIF-related adverse event.

## RÉSUMÉ

**CONTEXTE:**

La rifampicine (RIF) est généralement considérée comme le pilier du traitement de la TB, cependant, ses effets indésirables limitent fréquemment son utilisation.

**MÉTHODES:**

Dans cette étude de cohorte rétrospective nous avons examiné les patients traités pour la TB dans notre institution et avons comparé ceux qui ont reçu la RIF à ceux qui n'ont pas pu la tolérer.

**RÉSULTATS:**

Au total, 829 patients ont été inclus. Soixante-seize patients (9%) étaient intolérants au RIF. Les patients intolérants au RIF étaient significativement plus âgés (âge médian : 67 ans, IQR 50–78 vs. 48 ans, IQR 31–70 ; *P* < 0,0001), et étaient plus susceptibles d'être des femmes (57% vs. 41% ; *P* = 0,01) et d'avoir un diabète sucré concomitant (37,3% vs. 19% ; *P* < 0,0001) par rapport à ceux qui toléraient le RIF. L'intolérance au RIF était principalement due à une transaminite (25%), une cytopénie (14,5%), une éruption cutanée (17,1%) et une intolérance gastro-intestinale (7,8%). Vingt patients ont ensuite été soumis à un test de provocation à la rifabutine, avec un taux de succès de 70%. La durée moyenne du traitement était significativement plus longue chez les patients intolérants au RIF (335 vs. 270 jours ; *P* < 0.001). Aucune différence significative n’a été observée dans les résultats du traitement.

**CONCLUSION:**

L'intolérance au RIF est plus courante chez les patients plus âgés, les femmes et les patients atteints de diabète sucré. Les patients qui n'ont pas pu tolérer le RIF ont suivi un traitement plus long, mais cela n’a pas entrainé de différence dans les résultats du traitement. Lorsqu'elle a été tentée, la rifabutine a été bien tolérée par la plupart des patients ayant déjà présenté un effet indésirable lié au RIF.

According to the WHO, an estimated 10.6 million people were diagnosed with TB in 2021.^1^ TB continues to be a leading infectious cause of death worldwide with mortality rates of up to 50% if untreated. Adherence to a treatment regimen is critical for curing TB disease, limiting the spread of infection, and preventing the development of drug resistance.^2^

Rifamycins, a class of antibiotics including rifampicin (RIF), rifabutin (RFB) and rifapentine, are of significant importance in the treatment of TB given their substantial sterilising activity, which is critical to the prevention of relapse; they also have moderate early bactericidal activity and play an important role in the prevention of drug resistance.^3^ RIF is the most commonly used rifamycin and is part of the recommended standard first-line treatment regimen in combination with isoniazid, pyrazinamide and ethambutol. RIF inhibits bacterial RNA polymerase in a concentration dependent manner.^4^ Its use is often limited by drug–drug interactions via induction of the cytochrome *p450* enzyme system and *P*-glycoprotein multidrug efflux transporters,^5^ or by adverse effects, including dermatologic events, cytopenias, transaminitis and gastrointestinal intolerance. RFB has a similar mechanism of action, but has fewer drug–drug interactions,^6^ and has been widely used in patients with HIV co-infection. However, limited data are available regarding the use of RFB in patients who are intolerant of RIF.

Prior to the introduction of standardised shorter multidrug-resistant TB (MDR-TB) treatment regimens, the World Health Organization recommended a total treatment duration of 18–20 months in individuals with RIF-resistant TB,^7^ and clinical practice guidelines published by the American Thoracic Society/US Centers for Disease Control/European Respiratory Society/Infectious Diseases Society of America recommend a total treatment duration of 15–21 months for RIF-resistant/MDR-TB.^8^ Similarly, the Canadian Tuberculosis Standards recommend extending therapy to 12–18 months if a rifamycin is not included in the treatment of drug-susceptible TB.^3^ Although critically important to the TB treatment regimen, RIF intolerance if fairly common,^9^ resulting in exclusion from the TB treatment regimen and substantially longer treatment courses that are associated with greater toxicity and poorer adherence.^10,11^

In our tertiary TB referral centre, where many patients are elderly and medically complex, RIF intolerance is relatively common. We sought to compare characteristics and outcomes between patients who received RIF throughout treatment and those who were RIF-intolerant. We reviewed reasons for RIF discontinuation, identified risk factors for RIF intolerance, and evaluated the patients who were successfully treated with RFB in place of RIF.

## METHODS

West Park Healthcare Centre is a provincially designated treatment centre for TB and is one of three adult TB clinics in the city of Toronto, ON, Canada, which service a population of approximately 2.96 million people, and a broader population of 6.4 million in the Greater Toronto Area.^12^ Approximately 300 new cases of TB disease are reported each year in Toronto.^13^

We conducted a retrospective cohort study of all patients with TB disease treated at our institution between 1 January 2010 and 31 August 2020. The study was approved by the West Park Healthcare Centre Research Ethics Board, Toronto, ON, Canada.

### Study inclusion and exclusion criteria

We included patients who met the following criteria: age >18 years; diagnosis of TB disease (by nucleic acid amplification testing, culture, or clinical diagnosis); received RIF initially as part of the intended treatment regimen; and initiated treatment between 1 January 2010 and 31 August 2020. Patients were excluded from the study if they had RIF resistance or if they never received RIF as part of their initial treatment.

### Institutional approach to drug intolerance

We are a referral site for complex TB and as such, our patient population has significant co-morbid disease and medical complexity. Medications are frequently stopped and restarted in the early phases of treatment as a regimen is being established. Medications are taken on an empty stomach. When gastro-intestinal (GI) intolerance is encountered, antibiotics are divided over the day and anti-nausea medications are administered. For other adverse events, therapy is stopped and sequential rechallenge attempted until an alternative regimen is constructed. Minor drug toxicity is often overcome with supportive care during the drug rechallenge period. Once the culprit drug/drugs are identified, new drugs are introduced until the regimen is felt to be adequate by the treating physician.

### Definition of rifampicin intolerance

Patients were considered RIF intolerant if they received RIF for <8 consecutive weeks. Eight weeks was chosen as a cut-off since most adverse events with RIF are known to occur during the early weeks of treatment.^14^ Eight weeks is also the duration of the intensive phase of TB treatment and an important time point in TB treatment.

Alternative medications used included fluoroquinolones (moxifloxacin or levofloxacin), clofazimine, amikacin, para-aminosalicylic acid, cycloserine, ethionamide and linezolid. Bedaquiline could only be attained for use in MDR-TB in Canada during the study period and was therefore not used in this population.

### Data collection

Patient demographics at the time of treatment initiation, including age, weight, and country of birth/origin; clinical details including location of disease and comorbidities; and TB characteristics, including smear positivity and microbiologic features were retrospectively abstracted from patient charts. Subsequent treatment details including medications received, duration of each treatment, laboratory abnormalities, and patient reported adverse events over the course of treatment were also abstracted. Drugs given for >8 consecutive weeks were considered included as part of the treatment regimen. Clinical outcomes over a minimum 2-year follow-up period were recorded.

### Adverse events and clinical outcomes: definitions

Adverse events were defined as any clinical or laboratory abnormality requiring treatment alteration (at the discretion of the treating clinician). Blood work was monitored weekly in inpatients and at least monthly in outpatients. End of treatment outcomes were defined according to previous WHO definitions, and included success (cure/treatment completion), treatment failure, death during treatment, and loss to follow up/not evaluated.^15^ The definition for treatment failure was modified slightly from the WHO such that a regimen change due to adverse events alone was not considered a failure. All patients who had outcomes evaluated without loss to follow up were followed for a minimum of 2 years post-treatment completion to assess for relapse.

### Statistical analysis

Descriptive statistics were calculated for baseline variables and treatment characteristics. Categorical variables were expressed as number (percentage) and mean (standard deviation [SD]) using the χ^2^ test or Fisher’s exact test; continuous variables were expressed as median (interquartile range [IQR]) using the Mann–Whitney *U*-test. For binary treatment outcomes, exact binomial confidence intervals were reported. Risk factors associated with intolerance on univariate analysis were included in a logistic regression model. Statistical significance was set at *P* <0.05. All analyses were performed using Stata v15 (StataCorp, College Station, TX, USA).

## RESULTS

### Patient characteristics and treatment details

Patient characteristics are detailed in Table 1. Nine hundred and twelve patients had a diagnosis of TB and were screened for inclusion into the study; 75 patients had RIF resistance, and 8 patients were started directly on RFB due to expected drug-drug interactions. Therefore, over the study period, a total of 829 patients met inclusion criteria; 43% were female and 83% had pulmonary involvement.

**Table 1.** Baseline patient characteristics.

	RIF-tolerant	RIF-intolerant	*P*-value
	(*n* = 753)	(*n* = 76)	
	*n/N* (%)	*n/N* (%)	
Female sex	312/753 (41)	43/76 (57)	0.01
Age, years, median [IQR]	48 [31–70]	67 [50–78]	<0.001
BMI, kg/m^2^, median [IQR]	21.4 [18.8–24.6]	22.1 [18.9–24.6]	0.61
Region of origin			0.51
Africa	97 (12.9)	8 (10.5)	
Americas	130 (17.3)	5 (6.6)	
Europe	36 (4.8)	5 (6.6)	
South-East Asia	475 (63.2)	56 (73.7)	
Eastern Mediterranean	14 (1.9)	1 (1.3)	
Western Pacific	0	1 (1.3)	
TB location			
Pulmonary	404 (53.7)	48 (63.2)	
Extrapulmonary	133 (17.7)	10 (13.2)	
Both	216 (28.7)	18 (23.7)	
TB characteristics			
AFB smear-positive	360 (48.2)	39 (52)	0.25
Cavitary disease	234 (31.1)	25 (32.9)	0.75
Bilateral disease	334 (44.7)	42 (55.3)	0.08
Comorbidities			
HIV	15 (2.2)	3 (4.3)	0.28
Diabetes mellitus	143 (19.0)	28 (37.3)	<0.001
Smoking	136 (18.8)	6 (8.3)	0.08
Alcohol use	256 (35.3)	14 (19.4)	0.02

RIF = rifampicin; IQR = interquartile range; BMI = body mass index; AFB = acid-fast bacilli.

Seventy-six patients (9%) were intolerant to RIF. The median time to RIF discontinuation in those who were intolerant was 14 days (IQR 7–33). On univariate analysis, patients with RIF intolerance were significantly older (median age: 67 years, IQR 50–78 vs. 48 years, IQR 31–70; *P* < 0.0001), and were more likely to be female (57% vs. 41%; *P* = 0.01) and have concurrent diabetes mellitus (DM) (37.3% vs. 19%; *P* < 0.0001) than those who tolerated RIF. These associations remained significant on multivariate analysis. There was a higher rate of alcohol use in patients who tolerated RIF than those who did not (35.3% vs. 19.4%; *P* = 0.02) and a trend towards a higher rate of smoking in patients with RIF tolerance; however, this was not statistically significant.

Details of treatment are described in Tables 2 and 3. Table 2 outlines the medications used in patients who did not tolerate RIF. Isoniazid (68%), ethambutol (68%), a fluoroquinolone (moxifloxacin or levofloxacin) (53%) and pyrazinamide (45%) were most commonly used. The majority of patients (76%) were established on a 3 or 4 drug treatment regimen in the absence of RIF (Table 3).

**Table 2.** Drugs included in treatment regimens in patients with RIF intolerance.

Drugs included in treatment regimen (RIF-intolerant)	(*n* = 76)
Isoniazid	52 (68.4)
Ethambutol	52 (68.4)
Fluoroquinolone*	40 (52.6)
Pyrazinamide	34 (44.7)
Rifabutin	14 (18.4)
Clofazimine	9 (11.8)
Amikacin	6 (7.9)
Para-aminosalicylic acid	3 (3.9)
Cycloserine	3 (3.9)
Ethionamide	1 (1.3)
Linezolid	1 (1.3)

* Moxifloxacin or levofloxacin were used in all patients who received a fluoroquinolone.

RIF = rifampicin.

**Table 3.** Number of drugs included in treatment regimen in patients with rifampicin intolerance.*

Number of drugs used	(*n* = 57)
	*n* (%)
2	6 (10.5)
3	22 (38.6)
4	21 (36.8)
5	6 (10.5)
6	2 (3.5)

* Drugs included only if documented to have been given for >8 weeks consecutively.

### Adverse events

Adverse events attributed to RIF and RFB are outlined in Table 4. RIF intolerance was most commonly due to transaminitis (25%), followed by rash (17.1%), GI intolerance (7.8%) and thrombocytopenia (7.8%). Cytopenia (leukopenia, thrombocytopenia or pancytopenia) occurred in 11 patients (14.5%).

**Table 4.** Adverse events related to RIF and RFB use.

Adverse events leading to drug discontinuation	RIF	RFB
	(*n* = 76)	(*n* = 20)
	*n* (%)	*n* (%)
Transaminitis	19 (25)	1 (5)
Rash	13 (17.1)	2 (10)
Thrombocytopenia	6 (7.9)	2 (10)
GI intolerance	6 (7.9)	
DRESS syndrome	5 (6.6)	
Anaphylaxis	5 (6.6)	1 (5)
Hyperbilirubinemia	5 (6.6)	
Confusion	4 (5.3)	
Renal failure	3 (3.9)	
Drug interaction	3 (3.9)	
Leukopenia	3 (3.9)	
Pancytopenia	2 (2.6)	
Eosinophilia	1 (1.3)	
Hypertension	1 (1.3)	

RIF = rifampicin; RFB = rifabutin; GI = gastrointestinal; DRESS = drug reaction with eosinophilia and systemic symptoms.

Of the RIF-intolerant patients, 20 (26.3%) patients were challenged with RFB, and of these, 70% were challenged successfully (i.e., did not experience an adverse event prompting RFB discontinuation). RFB was not tolerated in 6 patients due to rash (*n* = 2), thrombocytopenia (*n* = 2), transaminitis (*n* = 1) and anaphylaxis (*n* = 1). Three patients had the same adverse event with RFB as that experienced with RIF (one patient with rash, one with thrombocytopenia and one with anaphylaxis).

### Treatment outcomes

The mean treatment duration was 270 days (SD ±187) in patients who tolerated RIF, compared to 335 days (SD ±200, *P* < 0.001) in those with RIF intolerance. There was no significant difference in treatment outcomes between the two groups (*P* = 0.52) (Table 5).

**Table 5.** Treatment outcomes.

	RIF-tolerant	RIF-intolerant	*P*-value
	*n* (%)	*n* (%)	
Total treatment duration, days, mean ± SD	270 ± 187	335 ± 200	<0.001
Treatment outcomes			
Success	522 (69.3)	48 (63.2)	0.52
LTFU/not evaluated	202 (26.8)	24 (31.6)	
Death	29 (3.9)	4 (5.3)	

RIF = rifampicin; SD = standard deviation; LTFU = loss to follow-up.

## DISCUSSION

Our study demonstrates that RIF intolerance is more common in older patients, in females, and in those with concurrent DM. Our findings are in keeping with a number of case series in the literature describing higher rates of medication-related adverse events in both drug-susceptible and MDR-TB in elderly patients,^16,17^ and in those with DM.^18,19^ Although TB is more commonly diagnosed in males, and males are known to have more unfavourable treatment outcomes and higher mortality,^20,21^ several studies suggest that drug intolerance is more commonly experienced by female patients.^22,23^ We found that females were more likely to have intolerance to RIF, but there was no difference in treatment outcomes between males and females in our cohort, indicating that alternative treatment regimens could be constructed despite RIF-related adverse events in our population. We found no significant difference in treatment outcomes between RIF-tolerant and RIF-intolerant patients, although those who were unable to tolerate RIF had a significantly longer duration of therapy. Although our sample size was limited, we found that when attempted, RFB was well tolerated in the majority of patients with a previous RIF-related adverse event.

RIF has excellent sterilising activity and is considered the backbone of standard TB treatment.^2^ Guidelines recommend prolonged courses of TB treatment in the absence of rifamycins. However, RIF intolerance is common, and historically, has been described in 2-4% of patients.^9^ A previous study conducted in New York City, NY, USA, assessed rates and risk factors of RIF discontinuation in patients with TB, and determined that while RIF was discontinued in 2% of patients, only 1% of discontinuations were done for appropriate reasons, including serious adverse reactions such as thrombocytopenia, kidney injury, hyperbilirubinemia or drug-induced rash/fevers.^24^ We had a substantially higher rate of RIF intolerance (9% of all patients), however as we are a tertiary TB centre, many of our patients are referred with complex disease, significant comorbidities or with already established drug intolerance. In our cohort, transaminitis, cytopenia, serious drug rash and refractory GI intolerance were the most common reasons for RIF to be discontinued. Similar to other studies in the literature, patients in our cohort who did not tolerate RIF typically received 3–4 drugs during their treatment, and had a prolonged treatment course.^14^

Treatment regimens for drug susceptible TB are significantly prolonged in the absence of rifamycin therapy, and there has been longstanding interest in using RFB in place of RIF due to better tolerability and fewer drug–drug interactions.^25,26^ Although a Cochrane review published in 2019 found no difference in adverse events between RFB and RIF,^27^ many case reports and case series in the published literature describe improved tolerability of RFB, and its successful use in place of RIF, including in the HIV-infected population,^28^ the elderly,^29^ and in those with prior adverse events with rifampin.^30–32^ A retrospective study published in 2011 examined a cohort of TB patients in Seattle, WA, USA, and out of 100 patients with an adverse effect from RIF, 57 were challenged with RFB. Of these, 72% completed treatment successfully with RFB. In this cohort, those with a dermatologic reaction to RIF were less likely to tolerate RFB.^33^ Our cohort excluded patients who were started on RFB at TB treatment initiation, and this included many of our HIV-infected patients and those with known RIF-related drug–drug interactions, including those on methadone and direct oral anticoagulants. In our group, 26% of patients with an adverse event to RIF were challenged with RFB, and this was successful in 70% of patients. Similar to our group, a cohort from Taiwan demonstrated that 72% of patients who were intolerant to RIF were able to tolerate RFB.^30^ In our cohort, 13 patients with a prior cutaneous reaction to RIF were challenged with RFB, and only a single patient had a similar reaction to RFB.

There are several limitations to our study. Over the 10-year study period, treatment varied widely in patients who did not tolerate first-line regimens. Our study was retrospective in nature. Because only a limited number of patients were challenged with RFB, we were unable to analyse for predictors of RFB intolerance in patients who were intolerant to RIF. Our treatment success rates were lower than those of other Canadian centres, which are reported to be >90%.^34^ There are several reasons for this. TB care in Ontario is decentralised, such that the majority of straightforward pulmonary TB is treated in the community. As we are a tertiary care centre for TB, patients referred to our site tend to be more medically complex with significant comorbid illness, higher rates of drug resistance/intolerance, and more disseminated/multi-organ disease. Once patients are established on appropriate treatment, their care is often transferred back to the original care provider, leading to a high number of lost to follow up outcomes in our cohort. Data on final drug treatment regimen and duration were consequently also missing for many patients.

We describe our experience with RIF intolerance in a low-incidence/high-resource setting. Our study highlights the high rate of adverse events from RIF in older patients, females, and those with DM, and adds to the limited but growing body of literature reporting successful use of RFB in the treatment of TB in patients who are intolerant to RIF. We believe that more patients can be challenged with RFB after RIF intolerance, as many of these patients will be able to successfully complete a rifamycin-based treatment regimen. Further research in this field including the use of prospective studies would be beneficial.
